# Using a quality improvement model to enhance providers’ performance in maternal and newborn health care: a post-only intervention and comparison design

**DOI:** 10.1186/s12884-017-1303-y

**Published:** 2017-04-12

**Authors:** Firew Ayalew, Gizachew Eyassu, Negash Seyoum, Jos van Roosmalen, Eva Bazant, Young Mi Kim, Alemnesh Tekleberhan, Hannah Gibson, Ephrem Daniel, Jelle Stekelenburg

**Affiliations:** 1 Jhpiego Ethiopia, Addis Ababa, Ethiopia; 2 Athena Institute, VU University, Amsterdam, The Netherlands; 3 Jhpiego, 1615 Thames St # 200, Baltimore, MD 21231 USA; 4 Department of Health Sciences, Global Health, University Medical Centre Groningen, University of Groningen, Groningen, The Netherlands; 5 Department of Obstetrics and Gynecology, Leeuwarden Medical Centre, Leeuwarden, The Netherlands

**Keywords:** SBM-R, Ethiopia, Antenatal care, Postnatal care, Labor and delivery, Provider performance, Quality improvement, Maternal health, Newborn health

## Abstract

**Background:**

The Standards Based Management and Recognition (SBM-R^©^) approach to quality improvement has been implemented in Ethiopia to strengthen routine maternal and newborn health (MNH) services. This evaluation assessed the effect of the intervention on MNH providers’ performance of routine antenatal care (ANC), uncomplicated labor and delivery and immediate postnatal care (PNC) services.

**Methods:**

A post-only evaluation design was conducted at three hospitals and eight health centers implementing SBM-R and the same number of comparison health facilities. Structured checklists were used to observe MNH providers’ performance on ANC (236 provider-client interactions), uncomplicated labor and delivery (226 provider-client interactions), and immediate PNC services in the six hours after delivery (232 provider-client interactions); observations were divided equally between intervention and comparison groups. Main outcomes were provider performance scores, calculated as the percentage of essential tasks in each service area completed by providers. Multilevel analysis was used to calculate adjusted mean percentage performance scores and standard errors to compare intervention and comparison groups.

**Results:**

There was no statistically significant difference between intervention and comparison facilities in overall mean performance scores for ANC services (63.4% at intervention facilities versus 61.0% at comparison facilities, *p* = 0.650) or in any specific ANC skill area. MNH providers’ overall mean performance score for uncomplicated labor and delivery care was 11.9 percentage points higher in the intervention than in the comparison group (77.5% versus 65.6%; *p* = 0.002). Overall mean performance scores for immediate PNC were 22.2 percentage points higher at intervention than at comparison facilities (72.8% versus 50.6%; *p* = 0.001); and there was a significant difference of 22 percentage points between intervention and comparison facilities for each PNC skill area: care for the newborn and health check for the mother.

**Conclusions:**

The SBM-R quality improvement intervention made a significant positive impact on MNH providers’ performance during labor and delivery and immediate PNC services, but not during ANC services. Scaling up the intervention to other facilities and regions may increase the availability of good quality MNH services across Ethiopia. The findings will also guide implementation of the government’s five-year (2015–2020) health sector transformation plan and health care quality strategies needed to meet the country’s MNH goals.

**Electronic supplementary material:**

The online version of this article (doi:10.1186/s12884-017-1303-y) contains supplementary material, which is available to authorized users.

## Background

Maternal and child mortality have declined over the past 20 years in Ethiopia. However, 2015 estimates show that mortality remains high in the months before and after childbirth, with 420 mothers dying per 100,000 live births, 41 infants dying per 1000 live births, 28 neonates dying per 1000 live births and neonatal deaths accounting for 47% of all under-five mortality [1]. In line with Sustainable Development Goals, by 2020 Ethiopia aims to reduce the maternal mortality ratio to 199 per 100,000 live births, the infant mortality rate to 20 per 1000 live births and the neonatal mortality rate to 10 per 1000 live births [2].

Achieving these goals will require improvements in the quality of maternal and newborn care services. The World Health Organization (WHO) defines eight standards for improving the quality of maternal and newborn care, one of which calls for “competent, motivated staff to be consistently available to provide routine care and manage complications” [3]. Multiple factors influence health workers’ performance, including health workers’ knowledge and skills gaps, caseload, patient demand, clinical practices, educational opportunities, availability of supplies and equipment and also the presence of a quality improvement process [4].

Various quality improvement models have been implemented in low, middle and high-income countries to improve healthcare providers’ performance and quality of services [5, 6]. WHO has concluded that any of these strategies can help to ensure high quality antenatal, intrapartum, and postnatal care [3].

Multifaceted interventions that include supervision, training, audit and feedback have been shown to have a greater impact than single interventions on the quality of health workers’ performance [5, 7]. One such approach is Standards-Based Management and Recognition (SBM-R^©^), which includes four stages to improve performance and quality of health services [8].

In Ethiopia, the Ministry of Health (MOH) began implementing the SBM-R quality improvement model in 2003 to improve HIV/AIDS and maternal and newborn health (MNH) services. However, the effect of SBM-R on healthcare providers’ performance and the quality of MNH services has not been evaluated in Ethiopia. This study seeks to address gaps in the literature regarding the effectiveness of MNH quality improvement interventions [3, 9] on providers’ performance by evaluating the impact of the SBM-R intervention in Ethiopia.

The evaluation asked: Do MNH providers in facilities that have implemented SBM-R perform better than providers at comparison facilities during routine delivery of antenatal care (ANC), uncomplicated labor and delivery, and immediate postnatal care (PNC) services within the first six hours after delivery [10]?

## Methods

### Study design and setting

This study employed a post-only intervention and comparison evaluation design to assess the impact of the SBM-R intervention on provider performance at public health facilities located in four major regions of Ethiopia: Tigray, Amhara, Oromia and Southern Nations, Nationalities and Peoples’ (SNNP). An SBM-R process to improve the performance and quality of MNH services was introduced at 116 government health facilities (15 hospitals and 101 health centers) from March 2011 to June 2014 with a phased-in approach. This study focuses on the 21 health facilities (15 health centers and 6 hospitals) in the first phase. All of these facilities had proceeded through the entire SBM-R process within three years of implementation and were ready for external assessment.

### Description of SBM-R intervention

Guided by national MNH policies and guidelines, performance standards in 10 technical areas were developed for providing quality MNH services, including 80 standards for health centers and 79 standards for hospitals. Technical areas included: routine ANC, uncomplicated labor and delivery care and post-partum care; management of antenatal, intrapartum, and postpartum complications; infection prevention; laboratory; pharmacy; human, physical and materials resources; information, education, communication and community participation; and management system. Each standard comprised a series of specific items that could be scored as a measure of achievement. After authorities endorsed the standards, they were introduced in each health facility. Health managers and providers received three rounds of SBM-R modular trainings focusing on how to use and implement the standards, measure progress, and recognize performance.

After completing the first round of SBM-R training, each facility established a quality improvement team to implement the intervention. This team conducted a baseline assessment of services at the facility using standard tools to identify performance gaps and analyze root causes. Commonly identified performance gaps included deficits in health providers’ skills and knowledge as well as shortages of supplies and equipment. Based on the initial gap assessment, intervention facilities received essential equipment and supplies (such as newborn weighing scales, autoclaves, examination beds, delivery beds, baby towels and hats, and infection prevention supplies), training on Basic Emergency Obstetric Newborn Care (BEmONC) for providers, and regular follow-up from regional health bureaus and partners, including site mentoring, review meetings and quarterly supportive supervision. Quality improvement teams at each facility conducted assessments every six months to track performance using direct structured observations, document review and provider interviews. Based on the results, the teams developed action plans to address remaining performance gaps. All target facilities completed the four stages of SBM-R and were eligible for external assessments verifying that they had achieved at least 80% of the standards.

### Sampling and sample selection procedure

Sample sizes were calculated for observations of providers’ performance in each of three service areas (ANC, labor and delivery, and immediate PNC) based on 95% level of statistical confidence, 80% statistical power, 20% expected difference in health providers’ performance between intervention and comparison groups and the recommended value of 1% intraclass correlation coefficient for median value of primary health care research [11]. The intraclass correlation coefficient is the correlation between responses given by randomly selected respondents within clusters or facilities to measure degree of similarity [12].

Health providers’ mean performance scores from a 2010 quality of care study in Ethiopia [13] informed the reference values used to calculate the three sample sizes. For ANC, a mean percentage performance score of 38% for preventive treatments, including the administration of tetanus toxoid and prescription or provision of iron/folic acid was used. For uncomplicated labor and delivery care, a mean percentage performance score of 29% on active management of the third stage of labor was used. For immediate PNC, a mean percentage performance score of 47% on immediate newborn care services was used. Based on these, we calculated sample sizes of 236 provider-client interactions (observations) for ANC, 226 for uncomplicated labor and delivery care, and 232 for immediate PNC. Each sample was divided equally between intervention and comparison groups.

A total of six hospitals and 15 health centers had completed all stages of SBM-R. Half (three hospitals and eight health centers) were randomly selected for the study sample. Comparison facilities that had not implemented SBM-R (three hospitals and eight health centers) were selected from the same region and zone as the intervention facilities, but from different districts. Comparison facilities were matched with intervention facilities on staff size and client volume, including the number of first ANC visits and deliveries attended by skilled birth attendants.

The estimated number of MNH providers (those who offer ANC, labor and delivery, and/or PNC services) at each facility was 12 per hospital and five per health center, for a total of 152 across all intervention and comparison facilities in the sample. Because the number of MNH providers at each facility was small, all of them were invited to participate in the study. Women who sought ANC or labor and delivery followed by immediate PNC at these facilities during the study period were invited to participate.

### Observation tool

Observations of service delivery using structured checklists were conducted to measure provider performance in ANC, uncomplicated labor and delivery care, and immediate PNC. The clinical observation tool listed essential maternity and newborn care tasks and was adapted from an earlier quality of care study in Ethiopia [13], national BEmONC training guidelines [10], and SBM-R tools. The ANC portion of the tool consisted of 53 tasks in eight skill areas; the labor and delivery portion consisted of 105 tasks in 10 skill areas; and the PNC portion included nine tasks in two skill areas. Observers coded each task as “Yes, task performed” or “No, task not performed.” The tool also captured basic characteristics of health providers.

### Data collection

Data were collected from July 7 to August 3, 2014 by 20 experienced assessors. Assessors consisted of midwives and health officers with at least a Bachelor of Science degree and national BEmONC master trainers. The assessors attended a four-day training on consent, study instruments, observation techniques, and data quality, and demonstrated their competency through role plays on consents and observation checklists before deployment. A team of two assessors conducted observations at each facility. Five supervisors and one study coordinator supervised them. To minimize observer’s bias, assessors were not assigned to health facilities where they worked.

Assessors collected information on providers’ socio-demographic characteristics and conducted observations while providers performed each step of routine ANC services, all stages of uncomplicated labor and delivery care, and/or the full range of immediate PNC services within the first six hours after delivery. Assessors stayed overnight at facilities to conduct observations, remaining up to five days at each facility until they observed at least one provider-client interaction per facility. Providers could be observed with more than one patient during this time. If no delivery took place in the facility during the assessors’ visit, the study coordinator instructed assessors to move to the next sample facility and observe more providers to compensate. Before traveling to the next sample facility, assessors and supervisors checked the completed observation tools for completeness and consistency.

### Data management and analysis

Data was cleaned and entered into CSPro V 5.0 and exported to STATA V 13.1 for statistical analysis. Descriptive statistics, including frequency and percentages, were computed to identify outliers and missing values. Data were nested in three levels: clients (first level) were clustered by provider (second level), and providers were clustered by facility (third level); this nesting structure creates dependency of data. The analysis examined three outcome variables: provider performance scores on ANC, uncomplicated labor and delivery care, and immediate PNC, calculated as the percentage of essential tasks in each service area completed by providers. For this study, health providers’ performance is defined as the extent to which actual work practices reflect the skills and knowledge taught in training using standardized operating procedures [14].

Multilevel analysis was used to calculate adjusted mean percentage performance scores and standard errors to compare intervention and comparison groups. The multilevel model is appropriate to generate adjusted standard errors of the estimates and test statistics or *p*-values for clustered data, instead of conventional linear models that overestimate test statistics and lead to misleading inferences [15, 16]. Mean performance scores ranged from 0 to 1. The mean percentage performance score was calculated by dividing mean performance scores by the maximum mean score and multiplying by 100.

Interclass correlation coefficients (ICC) were computed to assess variations explained by facility and health provider clustering effects on three outcome variables: health provider performance on ANC, uncomplicated labor and delivery care, and immediate PNC. ICC ranges from 0 to 1 and is used to estimate the degree of providers’ performance similarity within and among clusters, where an ICC value close to 1 indicates similarities between individual’s performance within clusters and an ICC value close to 0 shows independence of individual’s performance within clusters and/or an indication to use single level analysis [17]. ICC values suggest the use of multilevel analysis even if they are close to 0.05 [18].

A Chi-square test was used to assess associations of study participants and facility characteristics among intervention and comparison groups. A *p*-value of less than 0.05 was used for all statistical tests. We computed adjusted standard errors of the mean performance scores to calculate 95% of confidence interval of the mean.

### Ethical considerations

Study approvals were obtained from the Ethiopian Ministry of Science and Technology National Research Ethics Review Committee and the Johns Hopkins Bloomberg School of Public Health Institutional Review Board. Trained assessors informed health providers and women sought MNH services about the study and asked for their written consent to be observed. Woman was asked to allow trained assessor to observe only her labor and delivery services with the provider however, assessors did not collect data on her name, identification number and pregnancy history. For those women who arrived to the facility with labor pain, her next of kin was asked for written consent for observation. If a woman had complication, assessors did not ask written consent from a woman or her next of kin; and they waited another women who came for labor and delivery services to use for providers observations.

## Results

### Sample characteristics

The sample consisted of 147 MNH providers (79 in the SBM-R intervention group and 68 in the comparison group). Most providers were female in both intervention (77.2%) and comparison (70.6%) groups, and one-third were over age 30 (Table 1). A large majority were midwives and nurses, and about half worked at hospitals and half at health centers. Almost half had fewer than five years of work experience in both intervention (48.1%) and comparison (45.6%) groups. There were no significant differences in provider or facility characteristics between intervention and comparison groups.

**Table 1:** Characteristics of providers observed

Demographic and work-related characteristics	Intervention group (*n* = 79)		Comparison group (*n* = 68)		*p*-value (Chi square test)
	n	%	n	%	
Gender					
Female	61	77.2	48	70.6	0.360
Male	18	22.8	20	29.4	
Age					
< 25 years	23	29.1	27	39.7	0.337
25–30 years	28	35.5	18	26.5	
Over 30 years	28	35.4	23	33.8	
Qualification					
Nurse	29	36.7	28	41.2	0.746
Midwife	41	51.9	31	45.6	
Other (doctor, health officer)	9	11.4	9	13.2	
Years of work experience in the health care system (private and public)					
< 5 years	38	48.1	31	45.6	0.856
5 to 10 years	20	25.3	20	29.4	
> 10 years	21	26.6	17	25.0	
Type of facility					
Hospital	40	50.6	36	52.9	0.780
Health center	39	49.4	32	47.1	
Region					
Amhara	16	20.3	15	22.1	0.529
Oromia	25	31.7	24	35.3	
SNNP	22	27.8	12	17.6	
Tigray	16	20.2	17	25.0	

### Degree of similarity of MNH providers’ performance at facility and provider levels

As shown in Table 2, assessors observed 232 ANC clients who were attended by 63 different health providers, 226 labor and delivery clients who were observed with 117 different providers and 229 PNC clients who were observed with 109 providers. ICC values vary, but are higher in the intervention than in the comparison group with one exception (PNC services at the facility level). This suggests that the quality of services offered was more uniform at intervention than comparison facilities. The greatest differences between intervention and comparison groups were seen in labor and delivery services: provider performance was more similar within each facility in intervention (ICC = 0.775) than in comparison facilities (ICC = 0.454), and performance was also more similar for multiple observations of the same provider in intervention (ICC = 0.632) than in comparison facilities (ICC = 0.474).

**Table 2:** Number of MNH providers and provider-client interactions observed, and intraclass correlation coefficients

Service area	Number of MNH providers observed			Number of provider-client interactions observed			Intraclass Correlation Coefficient (ICC)		
	Total	Inter-vention group	Com-parison group	Total	Inter-vention group	Com-parison group	Cluster level	Inter-vention group	Com-parison group
ANC	63	29	34	232	118	114	Facility	0.551	0.458
							Provider	0.763	0.667
Labor & delivery	117	57	60	226	123	123	Facility	0.632	0.474
							Provider	0.775	0.454
PNC	109	57	52	229	119	110	Facility	0.530	0.573
							Provider	0.658	0.574

### Health provider performance

#### ANC services

Average overall performance scores for ANC services were 63.4% in the intervention and 61.0% in the comparison group (*p* = 0.650). There were no significant differences between study groups in any of the eight skill areas, although performance scores were higher by over 10 percentage points for rapid initial assessment, preventive treatment and counseling on danger signs in intervention as compared to comparison facilities. Health providers’ strengths and weaknesses, as evidenced by higher and lower scores in the various skill areas, were similar in both study groups (Table 3).

**Table 3:** Adjusted mean provider performance scores, by service area and study group

Skill areas	Number of items	Intervention group		Comparison group		% point difference between study groups	*p*-value^a^
		Mean score	Standard error	Mean score	Standard error		
Antenatal care (*n* = 124 for intervention group; *n* = 120 for comparison group)							
Rapid initial assessment	6	56.5	0.07	40.8	0.07	+15.7	0.099
Preventive treatment	3	58.8	0.05	47.7	0.06	+11.1	0.140
Counseling on danger signs	6	69.7	0.06	59.1	0.05	+10.6	0.174
Friendly reception for women	2	58.6	0.07	50.0	0.07	+8.6	0.346
Obstetrical history taking	11	81.8	0.07	77.4	0.07	+4.4	0.651
Laboratory screening tests	3	82.6	0.10	79.0	0.10	+3.6	0.783
Physical examination	11	73.8	0.03	70.4	0.03	+3.4	0.380
Birth preparedness	11	45.2	0 .07	43.5	0.07	+1.7	0.858
*Overall (composite) score*	*53*	*63.4*	*0.04*	*61.0*	*0.04*	*+2.4*	*0.650*
Labor and delivery (*n* = 123 for intervention group; *n* = 117 for comparison group)							
Rapid initial assessment	12	60.6	0.05	42.8	0.05	+17.8	**0.019**
Care during labor	10	81.1	0.03	66.0	0.03	+15.9	**0.001**
Assisting women to have safe and clean birth	13	58.9	0.04	43.7	0.04	+15.2	**0.003**
Immediate newborn care	4	76.9	0.04	61.9	0.04	+15.0	**0.013**
Preparation of supplies and equipment	13	81.7	0.03	67.8	0.03	+13.9	**0.001**
Physical and vaginal exam	18	78.1	0.04	65.2	0.04	+12.9	**0.013**
AMTSL^b^	7	82.9	0.03	71.7	0.03	+11.2	**0.005**
Obstetrical history taking	8	84.9	0.06	74.6	0.06	+10.3	0.240
Quality utilization of partograph	14	77.1	0.04	70.7	0.04	+6.4	0.256
Infection prevention steps	6	92.6	0 .02	87.1	0.02	+5.5	0.104
*Overall (composite) score*	*105*	*77.5*	*0.03*	*65.6*	*0.03*	*+ 11.9*	***0.002***
Immediate postnatal care (*n* = 119 for intervention group; *n* = 110 for comparison group)							
Care for the newborn within first hour after birth	5	79.3	0.04	57.0	0.04	+22.3	**0.001**
Check mother’s health after birth	4	66.2	0 .06	44 .2	0.06	+22.0	**0.012**
*Overall (Composite) score*	*9*	*72.8*	*0.05*	*50.6*	*0.03*	*+ 22.2*	***0.001***

^a^Results after adjusting for clustering effects

^b^ *AMTSL* Active management of third stage of labor

#### Uncomplicated labor and delivery services

Average overall performance scores for uncomplicated labor and delivery services were significantly higher in the intervention than in the comparison group (77.5% versus 65.6%, *p* = 0.002). MNH providers at intervention facilities significantly outperformed those at comparison facilities in 7 of 10 skill areas. Differences between the study groups were greatest for rapid initial assessment (60.6% versus 42.8%, *p* = 0.019), care during labor (81.1% versus 66.0%, *p* = 0.001), and immediate newborn care (76.9% versus 61.9%, *p* = 0.013). Differences between the study groups were smallest (less than 10 percentage points) and not significant for quality utilization of partograph and infection prevention (Table 3).

#### Immediate PNC services

Average overall performance scores for immediate PNC services were significantly higher at intervention than at comparison facilities (72.8% versus 50.6%, *p* = 0.001). There was a significant difference of 22 percentage points between study groups in both skill areas: care for the newborn and health check for the mother (Table 3).

## Discussion

This evaluation found that the SBM-R quality improvement intervention in Ethiopia had a positive impact on MNH providers’ performance in two out of three service areas: uncomplicated labor and delivery care and immediate PNC. Notably, significant and positive impacts on certain important skills that can save the lives of mothers and newborns were found. Rapid initial assessment determines whether pregnant women need emergency care, while immediate newborn care can identify and address potentially life-threatening conditions for newborns. Similarly, active management of the third stage of labor is a proven obstetric intervention for the prevention and management of postpartum hemorrhage [19]. SBM-R also contributed to substantial improvements on postnatal health checks for mother and baby after delivery, which are important to avert key causes of maternal and newborn deaths such as asphyxia, bleeding and severe infection [20].

Several factors likely contributed to these improvements. First is BEmONC training conducted in response to initial results of the SBM-R facility assessments, which primarily targeted midwives working in labor and delivery rooms. Training emphasized clinical competencies of labor and delivery care and immediate PNC services and utilized both simulated and clinical settings. Second is the low percentage of institutional deliveries in Ethiopia at the time of the study [21]; the lighter workload may have allowed midwives to take time needed to properly follow SBM-R standards before, during and after delivery. Third, the SBM-R intervention facilitated mobilization of resources within and outside facilities to address performance gaps. Also, intervention facilities received medical equipment and supplies to improve quality of intra- and postpartum care; this likely increased providers’ confidence in their clinical skills.

Findings on labor and delivery care and PNC are generally consistent with evidence from other SBM-R quality improvement interventions directed at MNH services. For example, studies in Malawi and Tanzania showed significant improvements in PNC performance, such as essential newborn care, at sites that implemented SBM-R [8, 22, 23]. Assessments conducted in 11 low-resource countries in Africa and Latin America found evidence that SBM-R interventions not only caused substantial improvement in labor and delivery service delivery practices such as partograph use and active management of the third stage of labor, but also contributed to positive intermediate and long-term health outcomes, such as lower rates of episiotomy and postpartum hemorrhage and reductions in maternal complications and maternal, neonatal and under five mortality [8, 24–27]. However, systematic evaluations of SBM-R in Malawi and Afghanistan found no significant differences in providers’ performance in labor and delivery care between intervention and comparison facilities [8, 22].

In contrast to labor and delivery care and PNC, the intervention did not show a significant improvement in providers’ performance on ANC. Anecdotal evidence suggests that the lesser impact of the intervention on the quality of ANC performance could be related to providers’ qualifications. Unpublished supervision reports from the SBM-R roll-out in Ethiopia indicate that routine ANC is often provided by nurses because the number of midwives at public health facilities is limited. These nurses were trained on general nursing during their pre-service education and did not specialize in MNH. They also did not have the opportunity to receive BEmONC training informed by SBM-R standards. In addition, ANC was often provided to multiple women at the same time, which may have prevented nurses and midwives from meeting SBM-R standards. It is important moving forward to provide refresher training on ANC tasks to all providers who offer these services, including nurses.

Previous studies of the impact of SBM-R interventions on ANC performance have shown mixed results. For example, after SBM-R was introduced at Zambian Defence Force facilities, overall ANC performance increased at intervention sites compared to control sites, but the difference was not statistically significant [28]. Exactly the same pattern was shown in this study, suggesting that SBM-R intervention likely contributed to the wellbeing of maternal, fetal and neonatal health. Other evaluations found that SBM-R interventions improved providers’ performance in ANC services in Afghanistan, but not in Zambia and Malawi [8, 22]. Another study in Kenya and Afghanistan indicated that quality improvement interventions enhanced ANC services [19, 25].

As noted above, many of the practices targeted by this intervention have the potential to save lives. Findings from systematic reviews in low- and middle-income countries confirm that quality improvement interventions focusing on healthcare providers’ skills can have a direct impact on health outcomes [8]. For example, SBM-R interventions in West African countries have contributed to decreases in institutional rates of postpartum hemorrhage and maternal and neonatal deaths [27, 29]. This suggests that scaling up SBM-R could contribute to achieving national goals for the reduction of maternal and newborn mortality rates. This quality model could also contribute to achieving the Ethiopia MOH’s 2020 strategic aims to improve intermediate and long-term health outcomes, such as increasing coverage of four ANC visits from 68 to 95%, births attended by skilled health personnel from 60 to 90%, and postnatal coverage from 90 to 95% [2].

With regard to sustainability, officials who offered supportive supervision and monitored the SBM-R intervention helped in building local ownership of quality improvement strategies at the district and facility levels. Results of the analysis of ICC values suggest that SBM-R contributed to standardization of provider skills in delivering ANC, labor and delivery care and immediate PNC services within and among facilities. Additional activities, such as ongoing mentorship at each facility, may be needed to reinforce and sustain these gains in providers’ MNH skills and performance over time.

### Strengths and limitations

Strengths of the study included its reliance on direct observations of routine services by experienced assessors using comprehensive and verifiable tools to assess quality of care by comparing intervention and comparison groups. The biggest limitation is the lack of baseline information on provider performance, which makes it impossible to compare changes in provider performance over time between study groups. In future, pre-post study designs are needed to examine the full effects of SBM-R quality improvement tools. In addition, qualitative interview data that could provide a deeper understanding of providers’ performance and clients’ perspective on the quality of services are lacking. Findings cannot be generalized to all facilities across all regions, and it is possible that other quality improvement approaches fielded by international non-governmental organizations may have contaminated effects in comparison sites. Finally, the study may have introduced the Hawthorne effect, in which providers perform better than usual in response to being observed by external assessors; however, this would have affected both study groups equally.

## Conclusions

The SBM-R quality improvement intervention led to significant improvements in health providers’ performance in delivering uncomplicated labor and delivery care and immediate PNC services in Ethiopia, a country with limited resources and a low density of healthcare providers. This substantial change contributed to increased availability of good quality MNH services at intervention hospitals and health centers. It also strengthened key skills that have the potential to save lives and reduce maternal and newborn mortality.

The Ministry of Health integrated the SBM-R approach within the existing MNH performance and quality improvement tool; and suggests to scale up across the remaining health facilities in all regions of the country, in order to improve healthcare providers’ performance and the quality of care and to contribute to health system strengthening across Ethiopia. These findings will serve as input for implementation of the government’s five-year (2015–2020) health sector transformation plan and health care quality strategies to meet the country’s sustainable development goals.

## Additional file

Additional file 1: Consent form. (DOCX 40 kb)

## Abbreviations

AMTSL: Active management of third stage of labor
ANC: Antenatal care
BEmONC: Basic emergency obstetric and newborn care
ICC: Intraclass correlation coefficient
MNH: Maternal and newborn health
MOH: Ministry of Health
PNC: Postnatal care
SBM-R: Standards-Based Management and Recognition
SNNP: Southern Nations, Nationalities and Peoples
USAID: United States Agency for International Development
WHO: World Health Organization
