# Supplementary material for: Using a quality improvement model to enhance providers’ performance in maternal and newborn health care: a post-only intervention and comparison design
Source: BMC Pregnancy Childbirth. 2017 Apr 12;17:115. doi: 10.1186/s12884-017-1303-y (PMC5389001; doi:10.1186/s12884-017-1303-y)
Supplement: Additional file 1: — Consent form. (DOCX 40 kb) [file 12884_2017_1303_MOESM1_ESM.docx]

**CONSENT FORM**

**TO BE READ FOR STUDY PARTICPANTS**

**PURPOSE:** You are invited to take part in an evaluation study. Let me explain a little about the study.

**Who is conducting the Study:** Maternal and child health integrated program (MCHIP) is carrying out an evaluation of a quality improvement process at public health facilities. The Ministry of Health (MOH) has provided support for the study. I work for the MOH, but I don’t work at this facility and I am not from this community.

Purpose/Aims: The aim of this study is to evaluate the effects of the Standards-Based Management and Recognition (SBM-R) quality improvement process on the quality and utilization of maternal and newborn health services in Ethiopia.

**Health Facilities**: We will collect data at 22 health facilities. Half of the facilities have implemented the quality improvement (QI) intervention. The other facilities have not implemented the intervention.

**Participants**: In each of these facilities, all health providers responsible for maternal and newborn health services will be invited for observation

**PROCEDURES**

**What We Will Do**: In this study, we will observe your skills and interview you. The observations will take place while you are offering ANC, L&D, or PNC services. We will remain at this facility for several days and plan to observe all consultations. We are not sure exactly how many of your consultations/services we will observe. It may be as few as one or two consultations or as many as five or six consultations.

## In addition, we will interview you about your opinions of the work environment at this facility and your experience with the quality improvement intervention. During the interview, you may refuse to answer any questions that you do not wish to answer.

## Time Needed: This consent process takes about 10 minutes and the interview will take about 20 minutes. We can schedule the interview for a time that is convenient to you. We do not want to disrupt regular clinical care to patients/clients, or make patients/clients wait.

The clinical observations will take place while you are providing regular care. Therefore, they will not take up any extra time for you.

**Confidentiality of Data:** Both the observations and the interview will take place in a private room or space. No one else will be present. No one else will see or hear you during the interview. We will not record your name or official ID number at all. After collecting data, my colleague or I will put the data collection forms into a sealed envelope. We will keep the data confidential. We will not show the data to your supervisor. No one from this health facility or any other facility will see the data we collect. Your signed consent form will not be linked with your data in any way. When we return to the MCHIP office in Addis Ababa, our study team will enter the data into a computer database. In the final report, the results will be presented by groups of health facilities. We will not present the data of any single health facility. The final report will be shared with the MOH.

# RISKS/DISCOMFORTS: We understand that it may make you feel uncomfortable or nervous to be observed during your consultations and to answer questions about your workplace.

In any study, there may be a risk to providers’ jobs if the data are not kept confidential. However, we will take several measures to keep all data private and confidential. First, your name and ID number will not be written down anywhere. We will not show the data to your supervisor or anyone else. The completed data collection forms will be kept in sealed envelopes until they reach the MCHIP office in Addis Ababa.

# BENEFITS

Benefits to you

- You may receive no direct benefit from the study.
- You may get some satisfaction from knowing that the MOH is concerned about the situation at this health facility.
- In the future, the MOH may develop programs to improve providers’ skills at this health facility, but this is not guaranteed.

Benefits to Ethiopian society: The study contributes to improving the quality of care offered to pregnant women, new mothers, and their babies in Ethiopia.

# VOLUNTARY PARTICIPATION

## No Payment: You will not receive any payment or compensation. The health facility will also not receive any payment.

## Voluntary: You are free to participate or not. You do not have to answer any question that you do not want to. If you agree to participate, you can change your mind and stop participating at any time. If you decide not to participate, this will not affect your job at this health facility or in any other facility in any way.

**CONSENT**

I have read this consent form (or it has been read to me). All my questions about the study and my part in it have been answered. I freely consent to be in this study.

I authorize the use and disclosure of my health information to the parties listed in the authorization section of this consent for the purposes described above.

By signing this consent form, I have not given up any of my legal rights. Subject name:_____

Signature of Subject (18 years and older)________________ Date__________

Signature of Person Conducting Informed Consent Discussion_________ Date__________

# PERMISSION TO PROCEED

May I have your permission to proceed with the study? Agree [ ] Refused [ ]
